# Proviral HIV DNA sequencing to inform suitability for long-acting injectable cabotegravir/rilpivirine

**DOI:** 10.1097/QAD.0000000000004040

**Published:** 2024-12-05

**Authors:** Jasmini Alagaratnam, Niall Kelly, Simon Dustan, Nadia Naous, Modest Nwanbila, Ruth Byrne, Nicolo Girometti, David Asboe, Graham P. Taylor, Marta Boffito

**Affiliations:** a Department of HIV Medicine & Sexual Health, Chelsea & Westminster Hospital NHS Foundation Trust; b Section of Virology, Department of Infectious Disease, Faculty of Medicine, Imperial College London; c National Centre for Human Retrovirology, Imperial College Healthcare NHS Trust, London, UK.

## Abstract

Increasingly, people attending HIV services are requesting long-acting injectable (LAI) antiretroviral treatment (ART). However, without HIV RNA resistance-associated mutation results, individuals are considered unsuitable for LAI ART. We present our experience of sequencing proviral HIV DNA in 30 individuals to inform suitability for LAI ART, of whom 23 were considered suitable. In conclusion, optimization of diagnostic tools such as proviral HIV DNA sequencing to confirm suitability for LAI ART would be a welcome addition.

Long-acting cabotegravir and rilpivirine (CAB/RPV LA) is the first injectable ART approved to treat people with HIV in whom HIV replication is fully suppressed (plasma HIV-1 RNA <50 copies/ml) on a stable ART regimen without present or past evidence of viral resistance to, and no prior virological failure with, agents of the nonnucleoside reverse transcriptase inhibitor (NNRTI) and integrase inhibitor (INI) classes [1–3].

Testing for drug resistance-associated mutations (DRAMs) in plasma HIV RNA is an established approach for informing treatment options for people with detectable viraemia [4–7]. However, in practice, some people have no prior genotypic DRAMs report available, as these were not performed in the settings where they initiated ART. Once HIV is fully suppressed, it is not possible to test genotypically for HIV DRAMs using standard drug resistance assays. This presents a clinical conundrum because without documented evidence of the absence of resistance to cabotegravir and/or rilpivirine, individuals may not be considered suitable for a switch to CAB/RPV LA. This results in unfair penalization and inequitable access to injectable ART and the benefits associated with this such as support with poor adherence to oral medication, pill fatigue, and stigma.

Therefore, there is growing interest in investigating the clinical utility of testing for DRAMs in intracellular proviral HIV DNA [8] to inform treatment selection for people who are virologically suppressed and who may benefit from a treatment switch to injectable ART due to issues such as pill burden, adherence, stigma, safety and tolerability, drug-drug interactions, or individual preference.

Since the introduction of CAB/RPV LA into clinical practice in England, our clinical service has been utilizing proviral HIV DNA sequencing in people who are considering a switch to injectable CAB/RPV LA treatment, are virologically suppressed, have no history of NNRTI or INI failure but have no HIV RNA genotypic DRAMs result to support treatment decisions by individuals with their clinicians. Here, we report our experience; this retrospective observational project was approved by the Clinical Governance Committee of Chelsea and Westminster NHS Foundation Trust.

Between November 1, 2021, and February 29, 2024, of 236 individuals who were referred to the HIV multidisciplinary team meeting at Chelsea & Westminster Hospital NHS Trust for consideration of CAB/RPV LA, 30 individuals had proviral HIV sequencing performed to inform suitability for CAB/RPV LA between the January 31, 2023, and January 17, 2024; these individuals were otherwise eligible to switch to CAB/RPV LA based on the existing criteria [1]. Proviral HIV sequencing was performed at the Molecular Diagnostic Unit, Imperial College London, using the MiSeq platform (Illumina, San Diego, California, USA). DRAMs were interpreted according to the online HIV database (HIVdb) program from Stanford University [9]. All 30 individuals had plasma HIV RNA less than 50 copies/ml at the time of proviral HIV sequencing. Of these, 23 individuals were considered suitable to switch to CAB/RPV LA, as no major or minor DRAMs or HIV clade contraindications were identified in proviral HIV sequences (Table 1). To date, 19 of these 23 individuals have initiated CAB/RPV LA and have been on CAB/RPV LA for a median of 244 days [interquartile range (IQR) 108–322] and all 19 individuals have maintained an undetectable plasma HIV viral load (<20 HIV RNA copies/ml). The median duration of full viral suppression in these individuals (excluding viral blips, i.e., a single episode of plasma HIV viral load between 50 and 200 HIV RNA copies/ml followed by undetectable plasma HIV viral load of <50 HIV RNA copies/ml) prior to commencing CAB/RPV LA was 109 months (interquartile range 67–142.5 months). HIV proviral sequences were not detected in one individual, while mutations associated with resistance to cabotegravir and/or rilpivirine were identified on HIV proviral sequencing in six individuals (Table 1); CAB/RPV LA was not initiated in these seven individuals.

**Table 1:** Baseline demographics table of individuals who had proviral HIV DNA sequencing performed to inform suitability for long-acting injectable cabotegravir and rilpivirine.

Demographic parameter, results are median (IQR) or total (%) as appropriate	All individuals who had proviral HIV DNA sequencing to inform suitability for CAB/RPV LAI, *n* = 30	Individuals considered suitable for CAB/RPV LAI based on the proviral HIV DNA sequencing result, *n* = 23	Individuals considered not suitable for CAB/RPV LAI based on RAMs detected during proviral HIV DNA sequencing or where proviral HIV DNA sequences were not detected, *n* = 7
Age	43 (34, 50)	43 (34, 52)	40 (37, 47)
Male	27 (90)	21 (91)	6 (86)
Female	3 (10)	2 (9)	1 (14)
Ethnicity			
- White	14 (47)	9 (39)	5 (71)
- Black	6 (20)	4 (17)	2 (29)
- Asian	3 (10)	3 (13)	0 (0)
- Other	2 (6)	2 (9)	0 (0)
- Not stated	5 (17)	5 (22)	0 (0)
Current CD4^+^ T-cell count (cells/μl)	695 (550, 887)	683 (554, 874)	707 (568, 876)
Current CD4:CD8 ratio	1.1 (0.6, 1.4)	1.0 (0.6, 1.5)	1.1 (0.7, 1.3)
Duration since HIV diagnosis, years	10.5 (7.0, 18.0)	11.0 (7.0, 18.0)	9.0 (7.0, 19.0)
Duration since ART initiation, years	8.0 (6.0, 17.0)	8.0 (6.5, 15.0)	9.0 (7.0, 19.0)
ART regimen when proviral HIV DNA sequencing performed			
- 2 NRTIs + Raltegravir	3 (10)	2 (8)	1 (14)
- 2 NRTIs + DTG or BTG	11 (37)	7 (30)	4 (58)
- DTG + 3TC	3 (10)	2 (8)	1 (14)
- 2NRTIs + NNRTI	12 (40)	11 (50)	1 (14)
- 2 NRTIs + bPI	1 (3)	1 (4)	0
Clade			
- B	18 (60.0)	14 (60.8)	4 (57.1)
- C	3 (10.0)	2 (8.7)	1 (14.3)
- B and C	2 (6.7)	2 (8.7)	0
- A and D	1 (3.3)	1 (4.4)	0
- CRF01_AE	3 (10.0)	2 (8.7)	1 (14.3)
- CRF02_AG	2 (6.7)	2 (8.7)	0 (14.3)
- CRF06_cpx	1 (3.3)	0	1
RAMs listed for each individual as detected on proviral HIV DNA sequencing, *n* = 6			Individual 1 (Clade B): E138A Individual 2 (Clade CRF01_AE): G163GR Individual 3 (Clade C): G163GR Individual 4 (Clade B): K103KN, Y188YH Individual 5 (Clade CRF06_cpx): S68N, M230MI Individual 6 (Clade B): Y115YFIN, V106X

3TC, lamivudine; ART, antiretroviral treatment; bPI, boosted protease inhibitor; BTG, bictegravir; CAB/RPV LAI, cabotegravir and rilpivirine long-acting injectable; DTG, dolutegravir; NNRTI, nonnucleoside reverse transcriptase inhibitors; NRTI, nucleos(t)ide reverse transcriptase inhibitor; RAMs, resistance-associated mutations.

The application of this molecular technique has widened access to and confidence in prescribing injectable ART, along with all the benefits that come with it. As previously demonstrated by Ellis *et al.*[10], proviral HIV genotypic sequencing may provide additional information to facilitate ART optimization in treatment-experienced people with HIV, without leading to virological failure and, instead, improve long-term ART tolerability and the person's quality of life.

However, because of the scarce clinical evidence on the concordance of HIV RNA and HIV DNA genotypic sequencing resistance test results, HIV DNA genotypic sequencing results should be interpreted with caution and correlated with as many additional clinical factors as possible, such as ART history, ART genetic barrier to resistance, epidemiology of resistance in the country where HIV was presumably acquired, use of HIV preexposure prophylaxis (PrEP), and other individual-specific factors.

HIV proviral HIV sequencing interpretation remains a hotly debated topic with evolving evidence. Proviral sequencing may detect DRAMs that are not detected in circulating virus, thus providing extra information on archived mutations, which can reappear should ART be stopped or changed.

Cutrell *et al.*[11] demonstrated that proviral HIV DNA rilpivirine-associated mutations were multifactorially associated with confirmed virologic failure following ART switch to CAB/RPV LA in the ATLAS and ATLAS-2 M studies when at least two risk factors were present. Until further data become available, all major cabotegravir and rilpivirine-associated DRAMs are currently considered clinically relevant and would preclude an individual from switching to CAB/RPV LAI. Evidence from real-world experience and the CARES study [12] demonstrates increasing support for the use of LAI CAB/RPV among people with accessory resistance-associated mutation(s). Our center continues to review the emerging data, but in the interim, we have opted to take a more conservative approach until additional evidence becomes available about whether this support for LAI CAB/RPV use can be extrapolated to people with accessory DRAMs including the G163R mutation in proviral sequences, as seen in our case series.

As proviral sequencing is conducted at low mutation-detection thresholds, results may be susceptible to artefactual detection of mutations arising from PCR error and APOBEC (apolipoprotein B mRNA editing catalytic polypeptide-like)-mediated hypermutations [13], which are difficult to interpret in clinical practice. It is more likely that single mutations at APOBEC susceptible sites are drug-induced, while multiple mutations at APOBEC sites are likely to be APOBEC-induced, reflecting clonal expansion of the cell harboring the mutated provirus. On the contrary, proviral assessment may result in false negatives with low frequency, historical DRAMs not detected by HIV DNA sequencing.

Armenia *et al.*[14] observed that in individuals on virologically suppressive ART, a shorter duration of previous virological suppression along with the detection of DRAMs in HIV DNA and low nadir CD4^+^ T-cell count were predictors of virological failure following ART switch. Further data are required to optimize the use of proviral sequencing to inform switches in people with fully suppressed HIV infection.

Social transformations in recent years have led to a dramatic rise in global migration, thus expanding the scope of responsibility of healthcare providers to adapt and to continue to deliver equitable access to healthcare. Efforts should be focused on increasing diagnostic equity to ensure that all people with HIV have access to the optimal ART options for them. At present, this is supported by current low levels of baseline DRAMs to INIs, which are the preferred third agent ART option in the major HIV antiretroviral guidelines [5–7,15], and by the increasing evidence that two-drug ART regimens are effective and well tolerated [16–21].

The role of HIV DNA sequencing in clinical practice remains unclear; however, a structured approach and commitment to optimize alternative diagnostic tools within the HIV field can contribute to better health-related quality of life for people with HIV [22] and to the achievement of the 2030 ambitions, including eliminating AIDS as a public health challenge [23] and getting toward zero new HIV transmissions [24]. This report provides additional information from our HIV center's experience of using proviral sequencing to inform suitability for CAB/RPV LA where previous plasma HIV RNA sequencing results are unavailable. Longer-term follow up of the individuals who initiated CAB/RPV LA based on the absence of DRAMs to cabotegravir and/or rilpivirine on proviral HIV sequencing compared to settings where proviral sequencing was not available will further inform the use of this approach.

## Acknowledgements

J.A. and M.B. conceptualized the idea for the project; S.D. and G.P.T. planned and performed the laboratory analyses (HIV proviral sequencing); N.K. and J.A. performed data collection and data analysis; J.A. wrote the first draft of the manuscript; all authors reviewed the draft manuscript for intellectual content and contributed to the final manuscript.

### Conflicts of interest

J.A. has received support to attend scientific conferences and has research grant funding from Gilead Sciences and has received speaker's fees from Merck Sharp & Dohme and Gilead Sciences. M.B. has received travel and research grants from and has been advisor/speaker for Janssen, Roche, ViiV, GSK, Merck Sharp & Dohme, Gilead, Pfizer, AZ, Moderna, and Sanofi. For the remaining authors, none were declared.
